# 2-{4-[5-(3-Pyrid­yl)-2*H*-tetra­zol-2-ylmeth­yl]phen­yl}benzonitrile

**DOI:** 10.1107/S1600536808017959

**Published:** 2008-07-09

**Authors:** Wei Dai, Da-Wei Fu

**Affiliations:** a Ordered Matter Science Research Center, College of Chemistry and Chemical Engineering, Southeast University, Nanjing 210096, People’s Republic of China

## Abstract

In the title compound, C_20_H_14_N_6_, there are two mol­ecules with similar conformations in the asymmetric unit. The pyridine and tetra­zole rings are nearly coplanar; they are twisted from each other by dihedral angles of only 8.7 (2) and 7.4 (2)°. The nearer benzene ring makes dihedral angles of 69.9 (2) and 88.5 (2)° with the tetra­zole ring in the two mol­ecules.

## Related literature

For the use of tetra­zole derivatives in coordination chemistry, see: Arp *et al.* (2000 ▶); Hu *et al.* (2007 ▶); Wang *et al.* (2005 ▶); Xiong *et al.* (2002 ▶).
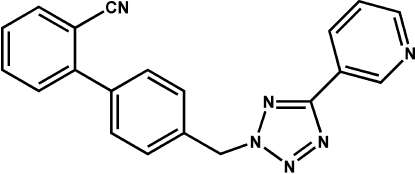


## Experimental

### 

#### Crystal data

C_20_H_14_N_6_
*M* *_r_* = 338.37
Triclinic, 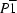

*a* = 10.2096 (9) Å
*b* = 13.3071 (16) Å
*c* = 13.709 (2) Å
α = 77.24 (2)°
β = 69.08 (2)°
γ = 83.52 (3)°
*V* = 1695.6 (4) Å^3^
*Z* = 4
Mo *K*α radiation
μ = 0.08 mm^−1^
*T* = 293 (2) K
0.4 × 0.35 × 0.35 mm

#### Data collection

Rigaku Mercury2 diffractometer
Absorption correction: multi-scan (*CrystalClear*; Rigaku, 2005 ▶) *T*_min_ = 0.958, *T*_max_ = 0.969
18012 measured reflections
8011 independent reflections
3834 reflections with *I* > 2σ(*I*)
*R*_int_ = 0.060

#### Refinement

*R*[*F* ^2^ > 2σ(*F* ^2^)] = 0.079
*wR*(*F* ^2^) = 0.239
*S* = 1.02
8011 reflections
469 parameters
H-atom parameters constrained
Δρ_max_ = 0.21 e Å^−3^
Δρ_min_ = −0.21 e Å^−3^

### 

Data collection: *CrystalClear* (Rigaku, 2005 ▶); cell refinement: *CrystalClear*; data reduction: *CrystalClear*; program(s) used to solve structure: *SHELXS97* (Sheldrick, 2008 ▶); program(s) used to refine structure: *SHELXL97* (Sheldrick, 2008 ▶); molecular graphics: *SHELXTL* (Sheldrick, 2008 ▶); software used to prepare material for publication: *SHELXTL*.

## Supplementary Material

Crystal structure: contains datablocks I, global. DOI: 10.1107/S1600536808017959/dn2354sup1.cif

Structure factors: contains datablocks I. DOI: 10.1107/S1600536808017959/dn2354Isup2.hkl

Additional supplementary materials:  crystallographic information; 3D view; checkCIF report

## Figures and Tables

**Fig. 1.**
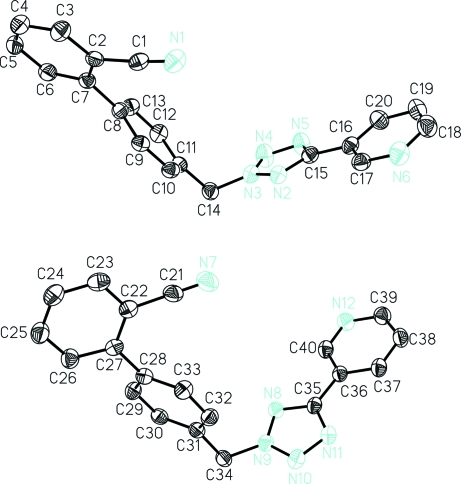
A view of the title compound with the atomic numbering scheme. Displacement ellipsoids were drawn at the 30% probability level. H atoms were omitted for clarity.

## supplementary crystallographic information

### Comment

In the past five years, our work have been focused on the chemistry of tetrazole derivatives because of their multiple coordination modes as ligands to metal ions and for the construction of novel metal-organic frameworks (Wang, *et al.* 2005; Xiong, *et al.* 2002). We report here the crystal structure of the title compound, 4-(4-((5-(pyridin-3-yl)-2*H*-tetrazol-2-yl)methyl)phenyl)benzonitrile.

The title compound contains two molecules with similar conformation in the asymmetrric unit. Each molecule is built up by four different rings (Fig.1). The pyridine and tetrazole rings are nearly coplanar and are only twisted from each other by a dihedral angle of 8.7 (2)° [7.4 (2)° for the second molecule]. The benzene ring makes a dihedral angle of 69.9 (2)° [88.5 (2)°] with the tetrazole ring owing to the methylene bridge which forces the two rings to be twisted from each other. The benzonitrile and the phenyl ring attached to it are twisted and make a dihedral angle of 46.5 (1)° [48.1 (2)°]. The C1—N1 and C21—N7 bond length of 1.153Å and 1.124Å conforms to the value for a C≡N bond. The bond distances and bond angles of the tetrazole rings are in the usual ranges (Wang *et al.*, 2005; Arp *et al.*, 2000; Hu *et al.*, 2007).

### Experimental

4-(4-((5-(Pyridin-3-yl)-2*H*-tetrazol-2-yl)methyl) phenyl)benzonitrile (3 mmol) was dissolved in ethanol (20 ml) and evaporated in the air affording colorless block crystals of this compound suitable for X-ray analysis were obtained.

### Refinement

All H atoms were fixed geometrically and treated as riding with C–H = 0.93Å (methine), 0.97 Å(methylene), with *U*_iso_(H) =1.2Ueq(C).

### Crystal data

**Table:**

C_20_H_14_N_6_	*Z* = 4
*M**_r_* = 338.37	*F*_000_ = 704
Triclinic, *P*1	*D*_x_ = 1.325 Mg m^−^^3^
Hall symbol: -P 1	Mo *K*α radiation λ = 0.71073 Å
*a* = 10.2096 (9) Å	Cell parameters from 3792 reflections
*b* = 13.3071 (16) Å	θ = 3.4–27.5º
*c* = 13.709 (2) Å	µ = 0.08 mm^−^^1^
α = 77.24 (2)º	*T* = 293 (2) K
β = 69.08 (2)º	Block, colourless
γ = 83.52 (3)º	0.4 × 0.35 × 0.35 mm
*V* = 1695.6 (4) Å^3^	

### Data collection

**Table:**

Rigaku Mercury2 (2x2 bin mode) diffractometer	8011 independent reflections
Radiation source: fine-focus sealed tube	3834 reflections with *I* > 2σ(*I*)
Monochromator: graphite	*R*_int_ = 0.060
Detector resolution: 13.6612 pixels mm^-1^	θ_max_ = 27.9º
*T* = 293(2) K	θ_min_ = 2.5º
ω scans	*h* = −13→13
Absorption correction: multi-scan(CrystalClear; Rigaku, 2005)	*k* = −17→17
*T*_min_ = 0.958, *T*_max_ = 0.969	*l* = −17→18
18012 measured reflections	

### Refinement

**Table:**

Refinement on *F*^2^	Secondary atom site location: difference Fourier map
Least-squares matrix: full	Hydrogen site location: inferred from neighbouring sites
*R*[*F*^2^ > 2σ(*F*^2^)] = 0.079	H-atom parameters constrained
*wR*(*F*^2^) = 0.239	*w* = 1/[σ^2^(*F*_o_^2^) + (0.1033*P*)^2^ + 0.086*P*] where *P* = (*F*_o_^2^ + 2*F*_c_^2^)/3
*S* = 1.02	(Δ/σ)_max_ = 0.033
8011 reflections	Δρ_max_ = 0.22 e Å^−^^3^
469 parameters	Δρ_min_ = −0.21 e Å^−^^3^
Primary atom site location: structure-invariant direct methods	Extinction correction: none

### Special details

**Table:**

Geometry. All esds (except the esd in the dihedral angle between two l.s. planes) are estimated using the full covariance matrix. The cell esds are taken into account individually in the estimation of esds in distances, angles and torsion angles; correlations between esds in cell parameters are only used when they are defined by crystal symmetry. An approximate (isotropic) treatment of cell esds is used for estimating esds involving l.s. planes.
Refinement. Refinement of *F*^2^ against ALL reflections. The weighted *R*-factor *wR* and goodness of fit *S* are based on *F*^2^, conventional *R*-factors *R* are based on *F*, with *F* set to zero for negative *F*^2^. The threshold expression of *F*^2^ > σ(*F*^2^) is used only for calculating *R*-factors(gt) *etc*. and is not relevant to the choice of reflections for refinement. *R*-factors based on *F*^2^ are statistically about twice as large as those based on *F*, and *R*- factors based on ALL data will be even larger.

### Fractional atomic coordinates and isotropic or equivalent isotropic displacement parameters (Å^2^)

**Table:**

	*x*	*y*	*z*	*U*_iso_*/*U*_eq_	
C1	0.2892 (3)	0.4722 (3)	−0.0251 (3)	0.0493 (8)	
C2	0.1973 (3)	0.5487 (2)	−0.0616 (3)	0.0468 (8)	
C3	0.1710 (4)	0.5407 (3)	−0.1523 (3)	0.0607 (9)	
H3	0.2188	0.4901	−0.1906	0.073*	
C4	0.0765 (4)	0.6058 (3)	−0.1859 (3)	0.0666 (10)	
H4	0.0610	0.6006	−0.2474	0.080*	
C5	0.0034 (4)	0.6799 (3)	−0.1279 (3)	0.0651 (10)	
H5	−0.0644	0.7228	−0.1487	0.078*	
C6	0.0313 (4)	0.6901 (3)	−0.0390 (3)	0.0573 (9)	
H6	−0.0172	0.7410	−0.0015	0.069*	
C7	0.1297 (3)	0.6266 (2)	−0.0042 (3)	0.0452 (8)	
C8	0.1637 (3)	0.6414 (2)	0.0893 (3)	0.0454 (8)	
C9	0.3033 (3)	0.6420 (2)	0.0825 (3)	0.0457 (8)	
H9	0.3748	0.6343	0.0193	0.055*	
C10	0.3369 (3)	0.6542 (2)	0.1689 (3)	0.0487 (8)	
H10	0.4306	0.6538	0.1627	0.058*	
C11	0.2337 (3)	0.6667 (2)	0.2634 (3)	0.0461 (8)	
C12	0.0941 (4)	0.6678 (3)	0.2696 (3)	0.0547 (9)	
H12	0.0227	0.6776	0.3321	0.066*	
C13	0.0601 (3)	0.6544 (3)	0.1838 (3)	0.0522 (8)	
H13	−0.0337	0.6542	0.1901	0.063*	
C14	0.2711 (4)	0.6775 (3)	0.3571 (3)	0.0555 (9)	
H14A	0.3618	0.7085	0.3311	0.067*	
H14B	0.2025	0.7239	0.3963	0.067*	
C15	0.3401 (4)	0.4363 (3)	0.4950 (3)	0.0514 (8)	
C16	0.4180 (4)	0.3383 (3)	0.5131 (3)	0.0513 (8)	
C17	0.5540 (4)	0.3219 (3)	0.4464 (3)	0.0570 (9)	
H17	0.5950	0.3757	0.3910	0.068*	
C18	0.5658 (5)	0.1567 (3)	0.5374 (4)	0.0750 (12)	
H18	0.6152	0.0940	0.5452	0.090*	
C19	0.4333 (5)	0.1661 (3)	0.6073 (3)	0.0731 (11)	
H19	0.3941	0.1112	0.6619	0.088*	
C20	0.3579 (4)	0.2580 (3)	0.5961 (3)	0.0635 (10)	
H20	0.2678	0.2662	0.6438	0.076*	
C21	0.6865 (4)	0.8056 (3)	0.0890 (3)	0.0586 (9)	
C22	0.6915 (3)	0.8914 (3)	0.0020 (3)	0.0507 (8)	
C23	0.6572 (4)	0.8719 (3)	−0.0823 (3)	0.0639 (10)	
H23	0.6367	0.8058	−0.0831	0.077*	
C24	0.6545 (4)	0.9530 (3)	−0.1641 (3)	0.0659 (10)	
H24	0.6351	0.9412	−0.2218	0.079*	
C25	0.6802 (4)	1.0505 (3)	−0.1611 (3)	0.0653 (10)	
H25	0.6748	1.1047	−0.2156	0.078*	
C26	0.7141 (4)	1.0698 (3)	−0.0781 (3)	0.0577 (9)	
H26	0.7322	1.1366	−0.0778	0.069*	
C27	0.7215 (3)	0.9901 (3)	0.0050 (3)	0.0465 (8)	
C28	0.7665 (3)	1.0123 (2)	0.0908 (2)	0.0457 (8)	
C29	0.7080 (3)	1.0970 (3)	0.1394 (3)	0.0510 (8)	
H29	0.6391	1.1393	0.1195	0.061*	
C30	0.7528 (3)	1.1180 (2)	0.2172 (3)	0.0477 (8)	
H30	0.7115	1.1732	0.2503	0.057*	
C31	0.8579 (3)	1.0579 (2)	0.2462 (3)	0.0478 (8)	
C32	0.9173 (4)	0.9740 (3)	0.1969 (3)	0.0523 (8)	
H32	0.9882	0.9330	0.2153	0.063*	
C33	0.8711 (4)	0.9519 (3)	0.1210 (3)	0.0534 (9)	
H33	0.9107	0.8954	0.0895	0.064*	
C34	0.9099 (4)	1.0827 (2)	0.3284 (3)	0.0540 (9)	
H34A	1.0023	1.1112	0.2935	0.065*	
H34B	0.8469	1.1337	0.3643	0.065*	
C35	0.8588 (4)	0.8580 (2)	0.5220 (3)	0.0466 (8)	
C36	0.7784 (4)	0.7789 (2)	0.6086 (2)	0.0457 (8)	
C37	0.8411 (4)	0.6860 (3)	0.6409 (3)	0.0571 (9)	
H37	0.9364	0.6726	0.6083	0.068*	
C38	0.7600 (4)	0.6138 (3)	0.7220 (3)	0.0641 (10)	
H38	0.8000	0.5512	0.7459	0.077*	
C39	0.6189 (4)	0.6359 (3)	0.7673 (3)	0.0646 (10)	
H39	0.5646	0.5857	0.8204	0.078*	
C40	0.6352 (4)	0.7937 (3)	0.6600 (3)	0.0538 (9)	
H40	0.5927	0.8555	0.6373	0.065*	
N1	0.3591 (3)	0.4065 (3)	0.0031 (3)	0.0677 (9)	
N2	0.3825 (3)	0.5112 (2)	0.4104 (2)	0.0522 (7)	
N3	0.2766 (3)	0.5798 (2)	0.4296 (2)	0.0531 (7)	
N4	0.1736 (3)	0.5504 (3)	0.5198 (3)	0.0775 (10)	
N5	0.2121 (3)	0.4598 (3)	0.5637 (3)	0.0717 (9)	
N6	0.6296 (4)	0.2346 (3)	0.4563 (3)	0.0729 (9)	
N7	0.6834 (4)	0.7392 (3)	0.1563 (3)	0.0806 (10)	
N8	0.8039 (3)	0.9433 (2)	0.4788 (2)	0.0511 (7)	
N9	0.9167 (3)	0.9889 (2)	0.4062 (2)	0.0499 (7)	
N10	1.0358 (3)	0.9351 (2)	0.4026 (2)	0.0596 (8)	
N11	1.0016 (3)	0.8513 (2)	0.4766 (2)	0.0554 (7)	
N12	0.5550 (3)	0.7260 (2)	0.7389 (2)	0.0621 (8)	

### Atomic displacement parameters (Å^2^)

**Table:**

	*U*^11^	*U*^22^	*U*^33^	*U*^12^	*U*^13^	*U*^23^
C1	0.0432 (18)	0.058 (2)	0.052 (2)	−0.0010 (16)	−0.0159 (17)	−0.0213 (17)
C2	0.0494 (19)	0.0420 (17)	0.049 (2)	−0.0119 (15)	−0.0147 (16)	−0.0065 (14)
C3	0.060 (2)	0.072 (2)	0.054 (2)	−0.0023 (19)	−0.0214 (19)	−0.0159 (18)
C4	0.075 (3)	0.071 (3)	0.062 (2)	−0.016 (2)	−0.034 (2)	−0.004 (2)
C5	0.063 (2)	0.063 (2)	0.073 (3)	−0.014 (2)	−0.037 (2)	0.011 (2)
C6	0.057 (2)	0.048 (2)	0.069 (2)	−0.0036 (17)	−0.026 (2)	−0.0077 (17)
C7	0.0377 (17)	0.0425 (17)	0.054 (2)	−0.0041 (14)	−0.0156 (16)	−0.0054 (15)
C8	0.0473 (18)	0.0410 (17)	0.050 (2)	0.0017 (14)	−0.0193 (16)	−0.0112 (14)
C9	0.0398 (17)	0.0434 (18)	0.051 (2)	−0.0025 (14)	−0.0103 (15)	−0.0126 (15)
C10	0.0397 (17)	0.0470 (18)	0.059 (2)	−0.0007 (14)	−0.0155 (17)	−0.0130 (16)
C11	0.0501 (19)	0.0379 (17)	0.051 (2)	−0.0005 (15)	−0.0190 (17)	−0.0081 (14)
C12	0.048 (2)	0.061 (2)	0.052 (2)	0.0051 (17)	−0.0118 (17)	−0.0169 (17)
C13	0.0389 (18)	0.058 (2)	0.062 (2)	0.0042 (15)	−0.0169 (17)	−0.0194 (17)
C14	0.065 (2)	0.0469 (19)	0.058 (2)	0.0020 (17)	−0.0252 (19)	−0.0119 (16)
C15	0.052 (2)	0.059 (2)	0.046 (2)	−0.0031 (17)	−0.0192 (17)	−0.0123 (16)
C16	0.054 (2)	0.058 (2)	0.049 (2)	0.0033 (17)	−0.0238 (18)	−0.0173 (16)
C17	0.056 (2)	0.056 (2)	0.064 (2)	−0.0023 (18)	−0.023 (2)	−0.0172 (18)
C18	0.086 (3)	0.061 (3)	0.082 (3)	0.010 (2)	−0.034 (3)	−0.019 (2)
C19	0.089 (3)	0.057 (2)	0.070 (3)	0.001 (2)	−0.029 (3)	−0.005 (2)
C20	0.067 (2)	0.060 (2)	0.059 (2)	−0.0051 (19)	−0.016 (2)	−0.0085 (18)
C21	0.068 (2)	0.049 (2)	0.064 (2)	−0.0112 (19)	−0.025 (2)	−0.0147 (19)
C22	0.0447 (19)	0.056 (2)	0.052 (2)	0.0004 (16)	−0.0148 (16)	−0.0159 (16)
C23	0.067 (2)	0.065 (2)	0.069 (3)	−0.009 (2)	−0.025 (2)	−0.027 (2)
C24	0.070 (3)	0.078 (3)	0.061 (2)	0.002 (2)	−0.031 (2)	−0.023 (2)
C25	0.075 (3)	0.068 (3)	0.059 (2)	0.002 (2)	−0.032 (2)	−0.0097 (19)
C26	0.064 (2)	0.053 (2)	0.060 (2)	0.0039 (18)	−0.027 (2)	−0.0118 (17)
C27	0.0409 (17)	0.054 (2)	0.048 (2)	−0.0014 (15)	−0.0146 (16)	−0.0164 (15)
C28	0.0450 (18)	0.0449 (18)	0.0462 (19)	−0.0030 (15)	−0.0126 (16)	−0.0112 (14)
C29	0.0481 (19)	0.052 (2)	0.055 (2)	0.0070 (16)	−0.0214 (17)	−0.0124 (16)
C30	0.055 (2)	0.0371 (17)	0.051 (2)	−0.0034 (15)	−0.0159 (17)	−0.0136 (14)
C31	0.0505 (19)	0.0456 (18)	0.0461 (19)	−0.0065 (15)	−0.0158 (16)	−0.0051 (15)
C32	0.051 (2)	0.0496 (19)	0.061 (2)	0.0011 (16)	−0.0245 (18)	−0.0132 (16)
C33	0.053 (2)	0.050 (2)	0.062 (2)	0.0094 (16)	−0.0221 (18)	−0.0230 (17)
C34	0.068 (2)	0.0457 (19)	0.052 (2)	−0.0100 (17)	−0.0259 (19)	−0.0027 (15)
C35	0.054 (2)	0.0444 (18)	0.050 (2)	0.0071 (16)	−0.0264 (17)	−0.0157 (15)
C36	0.057 (2)	0.0447 (18)	0.0433 (19)	0.0008 (15)	−0.0248 (17)	−0.0133 (14)
C37	0.059 (2)	0.053 (2)	0.062 (2)	0.0093 (18)	−0.0262 (19)	−0.0112 (18)
C38	0.079 (3)	0.048 (2)	0.070 (3)	0.007 (2)	−0.037 (2)	−0.0068 (18)
C39	0.075 (3)	0.062 (2)	0.058 (2)	−0.006 (2)	−0.027 (2)	−0.0074 (18)
C40	0.062 (2)	0.050 (2)	0.053 (2)	0.0082 (17)	−0.0256 (19)	−0.0136 (16)
N1	0.061 (2)	0.078 (2)	0.074 (2)	0.0183 (17)	−0.0294 (18)	−0.0337 (18)
N2	0.0522 (17)	0.0555 (17)	0.0503 (18)	0.0062 (14)	−0.0181 (14)	−0.0168 (14)
N3	0.0519 (17)	0.0600 (18)	0.0489 (18)	0.0058 (15)	−0.0190 (15)	−0.0148 (14)
N4	0.061 (2)	0.083 (2)	0.067 (2)	0.0175 (18)	−0.0082 (19)	−0.0046 (18)
N5	0.057 (2)	0.072 (2)	0.066 (2)	0.0097 (17)	−0.0089 (17)	0.0003 (17)
N6	0.074 (2)	0.073 (2)	0.075 (2)	0.0214 (19)	−0.0280 (19)	−0.0305 (19)
N7	0.112 (3)	0.062 (2)	0.076 (2)	−0.022 (2)	−0.039 (2)	−0.0089 (18)
N8	0.0549 (17)	0.0446 (15)	0.0557 (18)	0.0006 (13)	−0.0225 (15)	−0.0089 (13)
N9	0.0531 (17)	0.0520 (16)	0.0482 (17)	0.0003 (14)	−0.0219 (15)	−0.0105 (13)
N10	0.0548 (18)	0.072 (2)	0.0533 (18)	0.0049 (16)	−0.0218 (15)	−0.0122 (16)
N11	0.0589 (19)	0.0517 (17)	0.0562 (19)	0.0051 (14)	−0.0252 (16)	−0.0062 (14)
N12	0.0614 (19)	0.062 (2)	0.058 (2)	−0.0027 (16)	−0.0157 (17)	−0.0101 (16)

### Geometric parameters (Å, °)

**Table:**

C1—N1	1.153 (4)	C22—C23	1.404 (5)
C1—C2	1.437 (5)	C23—C24	1.379 (5)
C2—C3	1.391 (4)	C23—H23	0.9300
C2—C7	1.407 (4)	C24—C25	1.364 (5)
C3—C4	1.359 (5)	C24—H24	0.9300
C3—H3	0.9300	C25—C26	1.382 (5)
C4—C5	1.385 (5)	C25—H25	0.9300
C4—H4	0.9300	C26—C27	1.392 (4)
C5—C6	1.384 (5)	C26—H26	0.9300
C5—H5	0.9300	C27—C28	1.501 (4)
C6—C7	1.387 (4)	C28—C33	1.391 (4)
C6—H6	0.9300	C28—C29	1.402 (4)
C7—C8	1.499 (4)	C29—C30	1.391 (4)
C8—C13	1.381 (4)	C29—H29	0.9300
C8—C9	1.395 (4)	C30—C31	1.385 (4)
C9—C10	1.391 (4)	C30—H30	0.9300
C9—H9	0.9300	C31—C32	1.400 (4)
C10—C11	1.377 (4)	C31—C34	1.513 (4)
C10—H10	0.9300	C32—C33	1.382 (4)
C11—C12	1.395 (4)	C32—H32	0.9300
C11—C14	1.505 (4)	C33—H33	0.9300
C12—C13	1.391 (4)	C34—N9	1.465 (4)
C12—H12	0.9300	C34—H34A	0.9700
C13—H13	0.9300	C34—H34B	0.9700
C14—N3	1.460 (4)	C35—N8	1.324 (4)
C14—H14A	0.9700	C35—N11	1.366 (4)
C14—H14B	0.9700	C35—C36	1.462 (5)
C15—N2	1.324 (4)	C36—C37	1.385 (4)
C15—N5	1.361 (4)	C36—C40	1.390 (5)
C15—C16	1.469 (5)	C37—C38	1.376 (5)
C16—C20	1.387 (5)	C37—H37	0.9300
C16—C17	1.387 (5)	C38—C39	1.376 (5)
C17—N6	1.329 (4)	C38—H38	0.9300
C17—H17	0.9300	C39—N12	1.346 (4)
C18—C19	1.361 (6)	C39—H39	0.9300
C18—N6	1.364 (5)	C40—N12	1.326 (4)
C18—H18	0.9300	C40—H40	0.9300
C19—C20	1.379 (5)	N2—N3	1.323 (3)
C19—H19	0.9300	N3—N4	1.319 (4)
C20—H20	0.9300	N4—N5	1.308 (4)
C21—N7	1.122 (4)	N8—N9	1.326 (4)
C21—C22	1.448 (5)	N9—N10	1.330 (4)
C22—C27	1.394 (4)	N10—N11	1.316 (4)
			
N1—C1—C2	176.0 (3)	C25—C24—C23	120.6 (4)
C3—C2—C7	120.7 (3)	C25—C24—H24	119.7
C3—C2—C1	118.2 (3)	C23—C24—H24	119.7
C7—C2—C1	121.0 (3)	C24—C25—C26	120.9 (4)
C4—C3—C2	120.9 (4)	C24—C25—H25	119.5
C4—C3—H3	119.6	C26—C25—H25	119.5
C2—C3—H3	119.6	C25—C26—C27	120.6 (3)
C3—C4—C5	119.6 (4)	C25—C26—H26	119.7
C3—C4—H4	120.2	C27—C26—H26	119.7
C5—C4—H4	120.2	C26—C27—C22	117.7 (3)
C6—C5—C4	119.9 (4)	C26—C27—C28	119.4 (3)
C6—C5—H5	120.0	C22—C27—C28	122.9 (3)
C4—C5—H5	120.0	C33—C28—C29	118.4 (3)
C5—C6—C7	121.9 (4)	C33—C28—C27	120.8 (3)
C5—C6—H6	119.0	C29—C28—C27	120.7 (3)
C7—C6—H6	119.0	C30—C29—C28	120.1 (3)
C6—C7—C2	116.9 (3)	C30—C29—H29	119.9
C6—C7—C8	121.5 (3)	C28—C29—H29	119.9
C2—C7—C8	121.7 (3)	C31—C30—C29	121.1 (3)
C13—C8—C9	118.1 (3)	C31—C30—H30	119.5
C13—C8—C7	121.8 (3)	C29—C30—H30	119.5
C9—C8—C7	120.1 (3)	C30—C31—C32	118.7 (3)
C10—C9—C8	120.9 (3)	C30—C31—C34	121.1 (3)
C10—C9—H9	119.6	C32—C31—C34	120.1 (3)
C8—C9—H9	119.6	C33—C32—C31	120.3 (3)
C11—C10—C9	121.1 (3)	C33—C32—H32	119.9
C11—C10—H10	119.4	C31—C32—H32	119.9
C9—C10—H10	119.4	C32—C33—C28	121.3 (3)
C10—C11—C12	118.0 (3)	C32—C33—H33	119.3
C10—C11—C14	120.7 (3)	C28—C33—H33	119.3
C12—C11—C14	121.3 (3)	N9—C34—C31	109.9 (3)
C13—C12—C11	121.1 (3)	N9—C34—H34A	109.7
C13—C12—H12	119.5	C31—C34—H34A	109.7
C11—C12—H12	119.5	N9—C34—H34B	109.7
C8—C13—C12	120.8 (3)	C31—C34—H34B	109.7
C8—C13—H13	119.6	H34A—C34—H34B	108.2
C12—C13—H13	119.6	N8—C35—N11	112.3 (3)
N3—C14—C11	113.6 (3)	N8—C35—C36	124.8 (3)
N3—C14—H14A	108.8	N11—C35—C36	122.9 (3)
C11—C14—H14A	108.8	C37—C36—C40	117.6 (3)
N3—C14—H14B	108.8	C37—C36—C35	121.4 (3)
C11—C14—H14B	108.8	C40—C36—C35	121.0 (3)
H14A—C14—H14B	107.7	C38—C37—C36	119.0 (3)
N2—C15—N5	111.6 (3)	C38—C37—H37	120.5
N2—C15—C16	125.2 (3)	C36—C37—H37	120.5
N5—C15—C16	123.2 (3)	C39—C38—C37	118.8 (3)
C20—C16—C17	117.4 (3)	C39—C38—H38	120.6
C20—C16—C15	121.5 (3)	C37—C38—H38	120.6
C17—C16—C15	121.1 (3)	N12—C39—C38	123.7 (4)
N6—C17—C16	124.4 (4)	N12—C39—H39	118.1
N6—C17—H17	117.8	C38—C39—H39	118.1
C16—C17—H17	117.8	N12—C40—C36	124.7 (3)
C19—C18—N6	123.2 (4)	N12—C40—H40	117.7
C19—C18—H18	118.4	C36—C40—H40	117.6
N6—C18—H18	118.4	C15—N2—N3	102.3 (3)
C18—C19—C20	119.2 (4)	N4—N3—N2	113.4 (3)
C18—C19—H19	120.4	N4—N3—C14	122.7 (3)
C20—C19—H19	120.4	N2—N3—C14	123.8 (3)
C19—C20—C16	119.3 (4)	N5—N4—N3	106.7 (3)
C19—C20—H20	120.3	N4—N5—C15	106.0 (3)
C16—C20—H20	120.3	C17—N6—C18	116.4 (4)
N7—C21—C22	179.6 (4)	C35—N8—N9	102.1 (3)
C27—C22—C23	121.6 (3)	N8—N9—N10	113.6 (3)
C27—C22—C21	121.3 (3)	N8—N9—C34	123.2 (3)
C23—C22—C21	117.0 (3)	N10—N9—C34	122.8 (3)
C24—C23—C22	118.5 (3)	N11—N10—N9	106.5 (3)
C24—C23—H23	120.7	N10—N11—C35	105.6 (3)
C22—C23—H23	120.7	C40—N12—C39	116.1 (3)
